# Triple trauma, double uncertainty, and a singular imperative to address the mental health crises within asylum-seekers and refugees system: a commentary on Hvidtfeldt et al. (2021)

**DOI:** 10.1007/s00127-022-02318-7

**Published:** 2022-06-17

**Authors:** Sherifat Oduola, Jennifer Dykxhoorn

**Affiliations:** 1 School of Health Sciences, University of East Anglia, Norwich Research Park, Norwich, NR4 7TJ UK; 2 Division of Psychiatry, University College London, 149 Tottenham Court Road, London, W1T 7AD UK

The current geo-political crisis around the world is a stark reminder of the impact of war and displacement on the lives of individuals. In 2020, the UN Refugee Agency estimated that over 1% of the world’s population were forcibly displaced persons (82.4 million people) including 26.4 million refugees and 4.1 million asylum seekers [1]. Estimates suggest that the number of forcibly displaced people has continued to rise over the past year, reaching 84 million by mid-2021, and rising with ongoing conflicts in Syria, Afghanistan, South Sudan, Ukraine, and more. The rising number of forcibly displaced people adds urgency to ensuring the human rights, health, and dignity of those forced to leave their homes.

## Mental health problems for asylum seekers and refugees

People fleeing conflicts to seek refuge in other countries are at elevated risk of experiencing mental health problems due to exposure to traumatic events in their country of origin, during the asylum-seeking process, and resettlement in a host country. Many refugees face traumatic experiences, including war, torture, detention, deprivation of basic needs, loss of family and friends, and leaving home. Many experience further trauma during the asylum-seeking process as many face harrowing and dangerous journeys, unsafe conditions in refugee camps, lack of services, threats of detainment, and separation from family and friends. Finally, upon arrival into a host country and initiating an asylum claim, there are many challenges, from dealing with family separation, worry and guilt over those left behind, uncertainty of the future, financial stressors, lack of adequate housing and employment, high levels of social isolation, and facing anti-immigration rhetoric. Thus, forced displacement, the asylum-seeking journey, and the resettlement process can contribute to cumulative and chronic trauma for refugees (‘triple trauma’).

Supporting the mental health of asylum-seekers and refugees is a critical public mental health issue and estimating the burden of mental health problems remains an ongoing challenge. Hence, the article by Hvidtfeldt and colleagues [2] makes a welcome contribution to our understanding of how policies and administrative procedures can affect the mental health of asylum seekers. Using the Danish population registers, they compared over 6000 refugee fathers who had arrived alone and were later successful in reuniting with their spouses and children through family reunification. They showed that fathers waiting for their family faced an increased risk of mental disorders and that this risk increased as the length of family separation increased. This study provides clear evidence that any administrative delays in processing asylum claims can cause harm to an already vulnerable population. Currently, 78% of asylum-seekers in the UK wait more than 6 months for an initial decision, compared to 13% in 2014 [3]. Facing long wait times and chronic uncertainty about legal status can exacerbate the trauma that asylum seekers have already faced.

Those seeking family reunification face an additional wait after this initial decision for the outcome of their family reunification application, resulting in ‘double uncertainty’ and prolonged periods of family separation. Prolonged family separation during asylum-seeking contravenes the spirit of international refugee protections, which have been set up to ensure all people are afforded basic rights and freedoms. Instead of supporting vulnerable families who have been forcibly displaced, many asylum seekers face delays, which create prolonged periods of family separation. Forced family separation during asylum seeking has been shown to be a traumatic experience with long-lasting psychological impacts for parents and children [4]. Critically, protection from unnecessary family separation is enshrined in the UN Convention of Human Rights of the Child (Articles 9–10): “children must not be separated from their parents against their will unless it is in their best interests…” and that “governments must respond quickly and sympathetically if a child or their parents apply to live together in the same country…”[5]. These experiences of family separation and ongoing uncertainty about legal status can re-traumatise asylum-seekers and hinder recovery from previous traumatic experiences.

## Recommendations

In this paper, Hvidftfeldt and colleagues draw attention to how the health of asylum-seekers and refugees is the product of policies and administrative processes. With this in mind, we have made several recommendations for how host countries can better support the mental health of asylum seekers and refugees.

1. *First of all, do no harm—Re-examine asylum-seeking policies and procedures:* This research showed that the current system of asylum-seeking has the potential to introduce additional harm to people, particularly when prolonging family separation. From a public health perspective, these findings provide encouraging evidence that changing the asylum-seeking policies and systems may have a tremendous impact on the mental health of refugees. Providing timely decisions on asylum-seeking claims and responding swiftly to family reunification requests may be a powerful tool that supports the mental health of refugees and avoids introducing additional harm by extending the time spent in uncertainty and separated from family. Policies surrounding asylum decisions should be trauma-informed and take a rights-based approach. Rights-based approaches put asylum seekers at the centre of policies and ensure the human rights of all asylum-seekers are protected, including compassionate policies to ensure the timely reunification of refugee families.
2. *Support for resettlement:* The research by Hvidtfeldt and colleagues also demonstrated that the asylum-seeking process has long-lasting impacts, with those who had experienced long periods of family separation continuing to have elevated rates of mental disorders, even once family reunification was achieved. As social support and networks have been shown to reduce the risk of developing mental health problems, programmes that aid refugees in building social networks may be an important aspect of resettlement support. Further, ensuring access to trauma-informed, culturally appropriate care should be a priority. Access to medical care in the asylum-seeking process is critical for addressing health difficulties but also can be an opportunity for asylum-seekers to develop trust in health and social care. To ensure all asylum-seekers have to access appropriate care, programmes which support individuals in navigating health and social care may be required. Further research should explore what works to ameliorate the detrimental impact of family separation on later mental health.
3. *Data on the most vulnerable asylum seekers:* While this study by Hvidtfeld and colleagues is a welcome addition to the literature on the impact of asylum-seeking policies on mental health, this study was not able to include individuals whose asylum claim was denied or any undocumented migrants. These vulnerable asylum seekers may be at a particularly heightened risk of mental health problems, and more research is needed to understand the drivers of mental health problems in this group.

It is vital that we create systems which support refugees and asylum seekers which provide timely decisions on asylum-seeking claims, access to appropriate health and social care, and assistance in building social networks to reduce the burden of mental illness in asylum-seeking and refugee groups.
